# The Potential Neuroprotective Effect of *Cyperus esculentus* L. Extract in Scopolamine-Induced Cognitive Impairment in Rats: Extensive Biological and Metabolomics Approaches

**DOI:** 10.3390/molecules27207118

**Published:** 2022-10-21

**Authors:** Marwa M. Saeed, Álvaro Fernández-Ochoa, Fatema R. Saber, Rabab H. Sayed, María de la Luz Cádiz-Gurrea, Amira K. Elmotayam, Francisco Javier Leyva-Jiménez, Antonio Segura-Carretero, Rania I. Nadeem

**Affiliations:** 1 Lecturer of Pharmacology and Toxicology, Faculty of Pharmacy, Heliopolis University, Cairo 11785, Egypt; 2 Department of Analytical Chemistry, Faculty of Sciences, University of Granada, Avda Fuentenueva s/n, 18071 Granada, Spain; 3 Pharmacognosy Department, Faculty of Pharmacy, Cairo University, Cairo 11562, Egypt; 4 Department of Pharmacology and Toxicology, Faculty of Pharmacy, Cairo University, Cairo 11562, Egypt; 5 Department of Analytical Chemistry and Food Science and Technology, University of Castilla-La Mancha, Ronda de Calatrava, 7, 13071 Ciudad Real, Spain; 6 Regional Institute for Applied Scientific Research (IRICA), Area of Food Science, University of Castilla-La Mancha, Avenida Camilo Jose Cela, 10, 13071 Ciudad Real, Spain; 7 Department of Pharmacology and Toxicology, Faculty of Pharmacy, Heliopolis University, Cairo 11785, Egypt

**Keywords:** *Cyperus esculentus*, tiger nut, metabolomics, UHPLC-ESI-QTOF-MS/MS, foodomics, scopolamine, memory impairments

## Abstract

The aim of the present study is to investigate the phytochemical composition of tiger nut (TN) (*Cyperus esculentus* L.) and its neuroprotective potential in scopolamine (Scop)-induced cognitive impairment in rats. The UHPLC-ESI-QTOF-MS analysis enabled the putative annotation of 88 metabolites, such as saccharides, amino acids, organic acids, fatty acids, phenolic compounds and flavonoids. Treatment with TN extract restored Scop-induced learning and memory impairments. In parallel, TN extract succeeded in lowering amyloid beta, β-secretase protein expression and acetylcholine esterase (AChE) activity in the hippocampus of rats. TN extract decreased malondialdehyde levels, restored antioxidant levels and reduced proinflammatory cytokines as well as the Bax/Bcl2 ratio. Histopathological analysis demonstrated marked neuroprotection in TN-treated groups. In conclusion, the present study reveals that TN extract attenuates Scop-induced memory impairments by diminishing amyloid beta aggregates, as well as its anti-inflammatory, antioxidant, anti-apoptotic and anti-AChE activities.

## 1. Introduction

Alzheimer’s disease (AD) is a progressive neurologic disorder characterized by the presence of senile amyloid-beta (Aβ) plaques and neurofibrillary tangles leading to neurodegeneration. It is the most prevalent cause of dementia, accounting for about 60% of cases [1]. It is anticipated that the global burden of AD will rise to reach 106.8 million cases by 2050 [2]. AD patients suffer from a progressive loss of cognitive abilities resulting in memory and learning dysfunctions that are mainly correlated with declines in the cholinergic neurotransmission system [3]. Several factors have been implicated in the pathophysiology of AD including genetic mutations, Aβ accumulation, hyper-phosphorylation of tau protein, oxidative stress, mitochondrial dysfunction, inflammation and apoptosis. These factors likely act synergistically through complex interactions to promote neurodegeneration [4,5].

Scopolamine (Scop), a muscarinic cholinergic receptor antagonist, has been generally approved to induce memory deficits in experimental animals and to search for and evaluate anti-dementia drugs. Following Scop administration, the cholinergic neurotransmission is blocked and animals exhibit diminished cognitive performance [6]. Additionally, it has been reported that a Scop injection resulted in cognitive impairment together with changes in oxidative stress in the rat brain [7]. Recently, Tang et al., (2019) concluded that Scop is a useful pharmacological model simulating the pathological cellular alterations seen in AD patients and other AD models such as impaired antioxidative defense system, raised oxidative stress, mitochondrial dysfunction, apoptosis and neuroinflammation [3].

Until now, there has been no effective treatment for AD and currently available drugs exhibit minimal effect and poor control on the disease, targeting its late aspects with numerous side effects and fatal complications. These drugs slow down the progression of the disease and relieve the symptoms but at the same time fail to reach a definite cure [8]. Nowadays, there is a worldwide focus on the prospective of using natural herbs as neuroprotective agents.

Tiger nut (TN) (*Cyperus esculentus* L.) is a perennial plant belonging to the family Cyperaceae. The edible part of the plant is the spherical rhizome from the base of the tuber [9]. TN is known by other names such as Zulu nut, chufa (in Spanish), earth chestnut, yellow nutsedge, rush nut as well as ground almond [10]. It is consumed widely in western and eastern Africa and southern Europe, particularly Spain [11]. *Cyperus esculentus* L. is known in Egypt as “habb el ‘aziz” and it is one of the most ancient Egyptian crops [12]. It has been found in jars in pharaonic tombs [13]. TN can be eaten raw, roasted, soaked in water, baked or even as a refreshing drink known in Spain as tiger nut milk or horchata de chufas [9]. Several studies were performed to evaluate the nutritional value of TN and TN-derived products, revealing the benefits of consuming TN for general health and development for all ages. The tubers contain high amounts of carbohydrates and crude fibers. Studies have shown that the tubers are rich in sodium, phosphorus and calcium and show low levels of manganese, zinc, magnesium, copper and iron mineral contents [14]. They also contain vitamins A, C and E, together with several essential amino acids [15]. Further, TN oil is rich in oleic acid, palmitic and linoleic acids [16], which possibly mediate for its biological potential.

Plant phytochemicals vary across different matrices and exhibit a myriad of biological activities [17,18]. The phytochemical investigation of *Cyperus esculentus* L. revealed the presence of flavonoids, sterols, alkaloids, tannins and saponins [19]. These phytochemicals may be responsible for the various biological activities reported on tiger nuts, whether in folk uses or in different studies. The tubers are known to be used as diuretics, tonics, stimulants, emmenagogues and aphrodisiacs [15]. They are used to treat indigestion, flatulence, dysentery and diarrhea [20]. TN is used in the management of several pathophysiological conditions such as: coronary heart disease, hypercholesterolemia, colon cancer, obesity, diabetes, anemia and as an antimicrobial agent. Several reports showed that TN exhibits strong antioxidant activity [21]. This antioxidant activity may contribute to the managing of many oxidative stress-related diseases.

Several studies highlighted the implication of tiger nuts in biological health promotion and novel technological forms of foods in the Mediterranean region, together with the traditional uses of tiger nuts to improve memory and cognitive properties [22]. Nevertheless, the detailed phytochemical composition, or the neuroprotective mechanisms, of *Cyperus esculentus* L. have not been extensively explored. Therefore, our study aimed to investigate the phytochemical composition of *Cyperus esculentus* L., using UPLC-ESI-QTOF-MS analysis. Additionally, the potential neuroprotective effect of different doses of TN in Scop-induced memory deficit was assessed in rats in order to verify the putative mechanisms underlying this effect.

## 2. Results

### 2.1. Metabolite Profiling Using LC/MS

**Figure 1:**
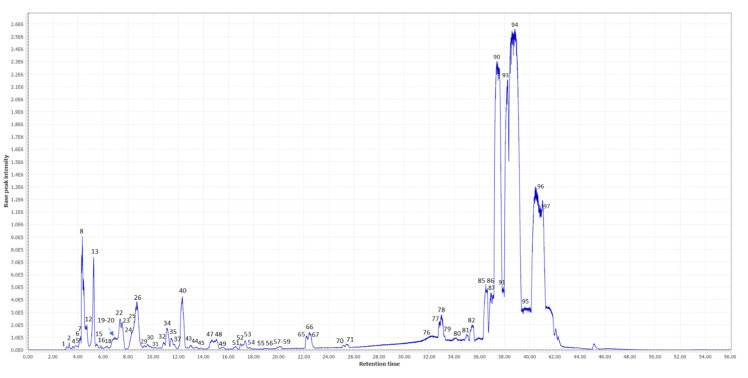
Base peak chromatogram (BPC) of the ethanolic extract of TN analyzed by UHPLC-ESI-QTOF-MS. The numbers refer to the dominant compounds in the BPC (see Table 1 for the tentative identity of these compounds).

The ethanolic extract of TN was analyzed by UHPLC-ESI-QTOF-MS with the aim to characterize the phytochemical composition that could be responsible for the neuroprotective potential. The base peak chromatogram (BPC) of that analysis is shown in Figure 1. After data processing, 97 potential molecular features were obtained and proposed for annotation. Information on these molecular features (retention time, *m*/*z*, formula molecular, ms/ms fragments, relative abundance, etc.) is shown in Table 1. According to the identification guidelines proposed by Sumner et al., [23], 58 compounds were annotated at level 2 by comparing their MS/MS spectra with those present in the databases, 30 solely based on their molecular formula and MS1 spectra (level 3) and 9 molecular features remained as unknowns (level 4).

Among the annotated compounds, phytochemicals belonging to the families of saccharides, amino acids, organic acids, fatty acids, phenolic compounds and flavonoids have been detected. In general, there is a high presence of fatty acids in the phytochemical composition of these matrices according to their relative abundances. This high presence of fatty acids, monounsaturated (e.g., oleic acid, eicosenoic acid, ricinoleic acid), polyunsaturated (e.g., linoleic acid, linolenic acid, docosahexaenoic acid) and saturated (e.g., stearic acid), agrees with previous studies that describe the relevance of fatty acids in Cyperus Esculentus Lativum [24]. Several of these fatty acids have been described as having an important role in regulating several processes within the brain and having neuroprotective properties [25]. Oleic acid (OA) has been detected with the highest relative abundance among the 97 compounds. The high concentration of this compound could be of great relevance, given that neuroprotective potential has been demonstrated for this compound. For instance, Song et al., (2019) carried out a study with OA in a rodent model of cerebral ischemia, showing the potential neuroprotective effects of this metabolite [26].

In addition, the presence of monosaccharides and oligosaccharides has also been detected in a high relative abundance. Different families of phenolic compounds have also been characterized, such as phenolic acids (ferulic acid, caffeoylquinic acid, etc.), flavonoids (e.g., apigenin, kaempferol, luteolin, etc.) and glycosylated flavonoids, etc. Although these compounds are found in lower relative concentrations, the neuroprotective potential of many of these compounds has been described in the literature [27,28,29].

Other families of compounds have also been detected, such as amino acids (tryptophan, arginine, phenylalanine, etc.), vitamins (pantothenic acid), nucleosides (guanosine, uridine, etc.) or organic acids (quinic acid and citric acid, etc.). Some of these have also been shown to play a role in neurodegenerative processes, such as tryptophan-related metabolites [30]. However, the relative abundance of these metabolites seems minor compared with the fatty acids detected. In any case, according to the literature, many of the annotated metabolites may contribute to the potential neuroprotective effect of this plant matrix, also considering possible synergistic effects between the different phytochemical compounds [31].

**Table 1:** The metabolite profiling of the ethanolic extract of *C. esculentus* L. rhizomes as analyzed by UHPLC-QTOF-MS.

N °	RT	*m*/*z* Experimental	*m*/*z* Theorical	Error (ppm)	Formula	Level of Annotation	Compounds	MS/MS Fragments	Rel. Ab. (%)	REF
1	3.11	201.0249	201.0260	5.47	C_5_H_6_N_4_O_5_	2	2-Oxo-4-hydroxy-4 carboxy-5-ureidoimidazoline	59/157	0.07	FDB001617
2	3.31	141.0164	141.0188	17.0	C_6_H_6_O_4_	2	Kojic acid	59/141	0.09	HMDB32923
3	3.52	131.0821	131.0821	0	C_5_H_12_N_2_O_2_	2	L-ornithine	70	0.03	[32]
4	3.80	173.1040	173.1039	0.58	C_6_H_14_N_4_O_2_	2	Arginine	131	0.1	[33,34]
5	3.82	195.0502	195.0505	1.53	C_6_H_12_O_7_	2	Gluconic acid	75/129/195	0.14	FDB001980
6	4.22	267.0717	267.0716	0.37	C_9_H_16_O_9_	3	xylo-manno-nononic acid ɣ-lactone	267	0.21	-
7	4.25	191.0553	191.0556	1.57	C_7_H_12_O_6_	2	Quinic acid	85/92/191	0.06	[35]
8	4.36	341.1082	341.1084	0.59	C_12_H_22_O_11_	2	Galactinol dihydrate	59/71/89/101/113/143/161	2.24	[32]
9	4.42	503.1607	503.1612	0.99	C_18_H_32_O_16_	3	Trisaccharide (raffinose)	503	0.17	[32], HMDB03213
10	4.50	337.0769	337.0771	0.59	C_12_H_18_O_11_	2	Ascorbyl glucoside isomer I	59/161/277/289	0.03	HMDB0253873
11	4.61	115.0031	115.0031	0	C_4_H_4_O_4_	2	Fumaric acid	71/87/99	0.02	[35]
12	4.72	341.1082	341.1084	0.59	C_12_H_22_O_11_	2	Sucrose	59/71/89/101/113/143/161	0.35	[32]
13	5.22	341.1082	341.1084	0.59	C_12_H_22_O_11_	2	Trehalose/maltose	129/143/161/179	1.37	[32]
14	5.30	133.0135	133.0137	1.50	C_4_H_6_O_5_	2	Malic acid	71/89/115/133	0.06	[35]
15	5.47	503.1607	503.1612	0.99	C_18_H_32_O_16_	3	Trisaccharide	503	0.12	[32]
16	5.82	503.1597	503.1612	2.98	C_18_H_32_O_16_	3	Trisaccharide	503	0.05	[32]
17	5.97	337.0763	337.0771	2.37	C_12_H_18_O_11_	2	Ascorbyl glucoside isomer II	59/161/277/289	0.03	HMDB0253873
18	6.32	251.0765	251.0780	5.97	C_10_H_12_N_4_O_4_	4	Unknown	251	0.11	-
19	6.61	191.0189	192.0270	0.60	C_6_H_8_O_7_	2	Citric acid	87 /111/129/173	0.03	[36]
20	6.76	295.1029	295.1029	0	C_11_H_20_O_9_	3	Aliphatic glucoside derivative	295	0.11	-
21	7.31	243.0621	243.0617	1.64	C_9_H_12_N_2_O_6_	2	Uridine	140/152/200	0.05	[37]
22	7.35	369.1404	369.1397	1.89	C_14_H_26_O_11_	3	Amylose	369	1	HMDB03403
23	7.56	295.1036	295.1029	2.37	C_11_H_20_O_9_	3	Aliphatic glucoside derivative	295	0.07	-
24	8.15	130.0868	130.0868	0	C_6_H_13_NO_2_	3	Leucine	130	1.10	[32,33]
25	8.34	329.0871	329.0873	0.60	C_14_H_18_O_9_	2	Dihydroxy benzoic acid methyl ester hexoside	125/153/167/270	0.06	[35]
26	8.69	413.1654	413.1659	1.21	C_16_H_30_O_12_	3	Glucopyranoside derivate	413	1.42	-
27	8.68	282.0843	282.0838	1.77	C_10_H_13_N_5_O_5_	2	Guanosine	133/150	0.03	FDB003632
28	8.95	493.1546	493.1557	2.23	C_20_H_30_O_1_4	2	O-hexosyl-O-methyl-myoinositol-dihydroxy benzoic acid	137/167/209/243/293/331	0.11	[35]
29	9.28	295.1027	295.1029	0.67	C_11_H_20_O_9_	3	Aliphatic glucoside derivative	295	0.11	-
30	9.54	383.1550	383.1553	0.78	C_15_H_28_O_11_	2	Butanediol apiosylglucoside	71/89/161	0.15	HMDB0033063
31	10.53	164.0710	164.0712	1.22	C_9_H_11_NO_2_	2	Phenylalanine	103/147/164	0.03	[32,33]
32	10.84	380.1545	380.1557	3.15	C_15_H_27_NO_10_	4	Unknown	380	0.2	-
33	10.89	218.1026	218.1028	0.91	C_9_H_17_NO_5_	2	Pantothenic acid	71/88/146	0.05	FDB008322
34	11.09	559.2228	559.2238	1.78	C_22_H_40_O_16_	3	Trisaccharide derivative	218	0.62	-
35	11.44	461.1652	461.1659	1.51	C_20_H_30_O_12_	2	Verbascoside	119/137/299	0.32	FDB018766
36	11.56	279.1078	279.1080	0.71	C_11_H_20_O_8_	3	Methyl glucopyranosyloxy butanoate	279	0.16	-
37	11.65	309.1186	309.1186	0	C_12_H_22_O_9_	3	Dideoxy-glucopyranosyl-ribohexose	309	0.15	-
38	11.84	359.0973	359.0978	1.39	C_15_H_20_O_10_	2	Glucosyringic acid	153/197/315/341	0.03	HMDB0303364
39	12.10	397.1657	397.1651	1.51	C_23_H_26_O_6_	2	Kanzonol M	176/161/181	0.02	HMDB0041101
40	12.34	397.1705	397.1710	1.26	C_16_H_30_O_11_	3	Glucopyranoside derivate	397	1.37	-
41	12.35	203.0823	203.0821	0.98	C_11_H_12_N_2_O_2_	2	Tryptophan	116/142	0.06	[32]
42	12.94	193.0504	193.0501	1.55	C_10_H_10_O_4_	2	Ferulic acid	107/134/149	0.05	[35,38]
43	12.98	503.1398	503.1401	0.59	C_21_H_28_O_14_	2	6-Caffeoylsucrose	149/161/179/323/341/443	0.12	FDB014172
44	13.45	353.0865	353.0873	2.26	C_16_H_18_O_9_	2	Chlorogenic acid	127/135/191	0.04	FDB002582 [37,39]
45	13.81	597.2177	597.2183	1.00	C_28_H_38_O_14_	4	Unknown	597	0.05	-
46	14.55	323.1337	323.1342	1.54	C_13_H_24_O_9_	4	Unknown disaccharide	323	0.12	-
47	14.67	293.1232	293.1236	1.36	C_12_H_22_O_8_	2	Ethyl-glucopyranosyl-butanoate isomer I	59/85/101/131	0.3	HMDB0031693
48	15.02	293.1232	293.1236	1.36	C_12_H_22_O_8_	2	Ethyl-glucopyranosyl-butanoate isomer II	59/85/101/131	0.32	HMDB0031693
49	15.54	351.1286	351.1291	1.42	C_14_H_24_O_10_	4	Unknown disaccharide	351	0.08	-
50	15.68	323.0976	323.0978	0.61	C_12_H_20_O_10_	4	Unknown disaccharide	323	0.04	-
51	16.58	609.1462	609.1456	0.98	C_27_H_30_O_16_	2	Luteolin-3′,7-di-O-glucoside	285/447	0.09	[40]
52	16.98	245.0923	245.0926	1.22	C_13_H_14_N_2_O_3_	2	cyclic 6-hydroxymelatonin	74/116/142/159/203/245	0.1	HMDB60810
53	17.32	245.0924	245.0926	0.81	C_13_H_14_N_2_O_3_	2	N-acetyl tryptophan	74/116/142/159/203	0.23	HMDB13713
54	17.67	683.1805	683.1823	2.63	C_30_H_36_O_18_	2	Rosmarinic acid di-O-hexoside	359/521	0.07	[41]
55	19.05	461.1080	461.1084	0.86	C_22_H_22_O_11_	2	Kaempferide 7-glucoside	283/269/299	0.05	HMDB38455
56	19.35	285.0395	285.0399	1.40	C_15_H_10_O_6_	3	Aureusidin	285	0.03	[40]
57	19.88	209.0792	209.0774	8.60	C_6_H_14_N_2_O_6_	4	Unknown	209	0.04	-
58	20.06	209.0790	209.0787	1.43	C_7_H_10_N_6_O_2_	4	Unknown nitrogenous compound	209	0.11	-
59	20.08	287.0560	287.0556	1.39	C_15_H_12_O_6_	2	Dihydrokaempferol	107/135/151/175/229/243	0.06	FDB012431
60	20.21	447.0923	447.0927	0.89	C_21_H_20_O_11_	2	Luteolin-O-glucoside	285	0.04	[40]
61	20.94	299.0488	299.0556	22.7	C_16_H_12_O_6_	3	Luteolin methyl ether	285	0.04	HMDB37339
62	21.27	269.0396	269.0450	20.1	C_15_H_10_O_5_	2	Apigenin	117/269	0.04	[37,40]
63	21.41	299.0556	299.0556	0	C_16_H_12_O_6_	3	Chrysoeriol	284/299	0.02	[34]
64	21.81	271.0555	271.0606	18.8	C_15_H_12_O_5_	2	Naringenin	107/119/151/177/217	0.04	[34]
65	22.22	285.0396	285.0399	1.05	C_15_H_10_O_6_	3	Kaempferol	285	0.23	[38]
66	22.42	285.0397	285.0399	0.70	C_15_H_10_O_6_	2	Luteolin	107/133/151/175	0.52	[36,38,40]
67	23.3	301.0714	301.0712	0.66	C_16_H_14_O_6_	2	Hesperitin	135/151/285	0.03	[42]
68	24.21	299.0556	299.0556	0	C_16_H_12_O_6_	2	Kaempferide	256/284	0.08	[34,43]
69	25.04	209.0815	209.0814	0.48	C_11_H_14_O_4_	3	Methylxanthoxylin	209	0.05	HMDB34047
70	25.39	299.0558	299.0556	0.69	C_16_H_12_O_6_	2	Isokaempferide	183/227/255	0.17	HMDB0302564
71	25.51	313.0713	313.0712	0.31	C_17_H_14_O_6_	2	Cirsimaritin	283/297/313	0.11	HMDB0250276
72	26.08	329.2324	329.2328	1.21	C_18_H_34_O_5_	2	Trihydroxy-octadecenoic acid	171/211/229/285/311	0.2	FDB002905
73	26.14	351.2144	351.2171	7.68	C_20_H_32_O_5_	4	Unknown	351	0.03	-
74	28.63	373.1291	373.1287	1.07	C_20_H_22_O_7_	3	Isohydroxymatairesinol	373	0.11	HMDB0301737
75	28.66	339.1235	339.1232	0.88	C_20_H_20_O_5_	3	Prenylnaringenin	339	0.09	HMDB0247465
76	31.09	313.2380	313.2379	0.32	C_18_H_34_O_4_	3	Octadecanedioic acid	313	0.05	HMDB00782
77	32.44	315.2525	315.2535	3.17	C_18_H_36_O_4_	3	Dihydroxyoctadecanoic acid	315	0.2	HMDB31008
78	32.95	205.1593	205.1592	0.48	C_14_H_22_O	3	2,4-di-tert-butylphenol	205	1.07	HMDB13816
79	33.15	295.2272	295.2273	0.33	C_18_H_32_O_3_	3	Hydroxylinoleic acid	277	0.25	HMDB0247599
80	34.60	199.1698	199.1698	0	C_12_H_24_O_2_	2	Dodecanoic acid	59/155	0.06	FDB030978
81	34.98	299.2592	299.2586	2	C_18_H_36_O_3_	2	Hydroxyoctadecanoic acid	255/269/281/299	0.52	FDB006898
82	35.40	297.2432	297.2430	0.67	C_18_H_34_O_3_	2	Ricinoleic acid	127/183/279	0.8	FDB012640
83	36.03	255.2329	255.2324	1.96	C_16_H_32_O_2_	3	Isopalmitic acid	255	0.18	[32]
84	36.27	281.2480	281.2481	0.35	C_18_H_34_O_2_	3	Elaidic acid	281	0.17	HMDB00573
85	36.50	277.2162	277.2168	2.16	C_18_H_30_O_2_	2	Linolenic Acid	119	2.27	[32]
86	36.70	227.2007	227.2011	1.76	C_14_H_28_O_2_	2	Myristic acid	209	0.77	[32]
87	36.89	253.2163	253.2168	1.97	C_16_H_30_O_2_	2	Palmitoleic acid	71/253	1.89	[32]
88	36.99	327.2315	327.2324	2.75	C_22_H_32_O_2_	2	DHA	229/283/309	0.92	HMDB0244316
89	37.38	581.4541	581.4570	4.99	C_38_H_62_O_4_	2	Oxygenated fatty acid derivatives	253/271	0.87	-
90	37.45	279.2337	279.2324	4.65	C_18_H_32_O_2_	2	Linoleic acid	71/261/279	17	[32]
91	37.95	267.2325	267.2324	0.37	C_17_H_32_O_2_	3	Heptadecenoic acid	267	0.35	HMDB31046
92	38.22	533.4538	533.4570	5.99	C_34_H_62_O_4_	2	Oxygenated fatty acid derivatives	293/533	0.77	-
93	38.26	255.2372	255.2324	18.8	C_16_H_32_O_2_	2	Palmitic acid	237	12	[32]
94	38.32	281.2480	281.2481	0.35	C_18_H_34_O_2_	2	Oleic acid	253/255/267	29.9	[32]
95	39.40	269.2483	269.2481	0.74	C_17_H_34_O_2_	3	Margaric acid	269	0.25	[32]
96	40.46	283.2640	283.2637	1.05	C_18_H_36_O_2_	2	Stearic acid	265	14.5	[32]
97	41.47	309.2792	309.2794	0.65	C_20_H_38_O_2_	3	Gondoic acid	309	0.32	[32]

### 2.2. Effect of Different Doses of TN on Scop-Induced Behavioral Changes in Rats

**Figure 2:**
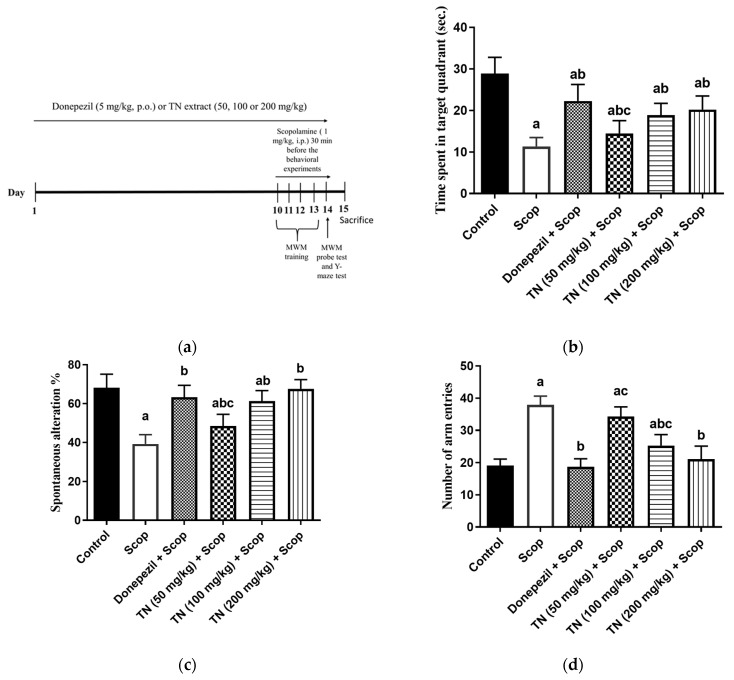
Experimental timeline (**a**). Effect of different doses of TN on the time spent in the target quadrant during the probe trial session in the MWM (**b**). Percentage of spontaneous alternation (**c**). Number of arm entries (**d**) in the Y maze test. Values are expressed as the mean ± SD (*n* = 15). Values are statistically significant at p < 0.05 versus the control group, *p* < 0.05 versus the Scop-treated group and *p* < 0.05 versus the donepezil-treated group.

In the MWM probe test, Scop-treated rats failed to recall the exact location of the platform, as evidenced by spending significantly less time in the target quadrant than the control rats by approximately 60.75%. On the other hand, the reduced time spent within the target quadrant by Scop-treated rats was significantly reversed by donepezil and TN 50, 100 or 200 mg by approximately 2-fold, 1.3-fold, 1.7-fold and 1.8-fold, respectively, demonstrating that TN ameliorated the Scop-induced deficiency in the memory of the animals (F (14, 79) = 57, *p* < 0.0001) (Figure 2b).

The administration of Scop revealed that short-term memory deficit manifested through a marked drop in the percentage of spontaneous alternation by approximately 42.53%, as well as a significant increment in the locomotor activity (the number of arm entries) by approximately 2-fold in the Y-maze test compared with the control rats. In contrast, compared with Scop, treatment with donepezil and TN 50, 100 or 200 mg significantly mitigated the decline of the spontaneous alternation percentage by approximately 1.6-fold, 1.2-fold, 1.6-fold and 1.7-fold, respectively (F (14, 84) = 61.98, *p* < 0.0001) (Figure 2c). Donepezil and TN 100 or 200 mg also decreased the locomotor activity by approximately 50.61%, 33.37% and 44.29%, respectively (F (14, 70) = 99.02, *p* < 0.0001) (Figure 2d).

### 2.3. Effect of Different Doses of TN on Scop-Induced Alterations in AChE Activity

**Figure 3:**
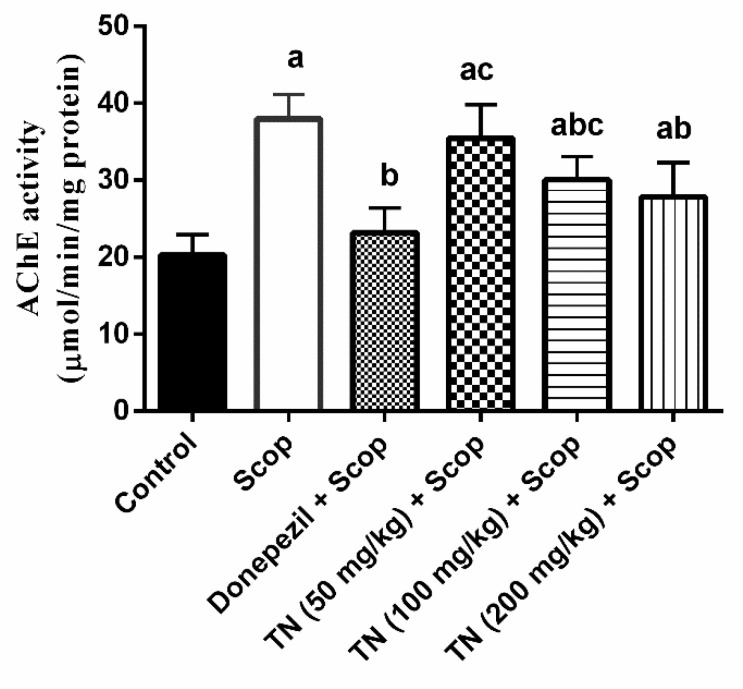
Effect of different doses of tiger nut on Scop-induced alterations in AChE activity. Values are expressed as the mean ± SD (*n* = 6). Values are statistically significant at *p* < 0.05 versus the control group, *p* < 0.05 versus the Scop-treated group and *p* < 0.05 versus the donepezil-treated group.

Rats injected with Scop exhibited a significant rise in hippocampal AChE activity by approximately 0.9-fold compared with the control group. Nevertheless, the administration of donepezil and TN 100 or 200 mg significantly suppressed the AChE activity (F (5, 30) = 22.51, *p* < 0.0001) by approximately 39.01%, 20.85% and 26.74%, respectively, compared with the Scop group values (Figure 3).

### 2.4. Effect of Different Doses of TN on Scop-Induced Oxidative Stress

**Figure 4:**
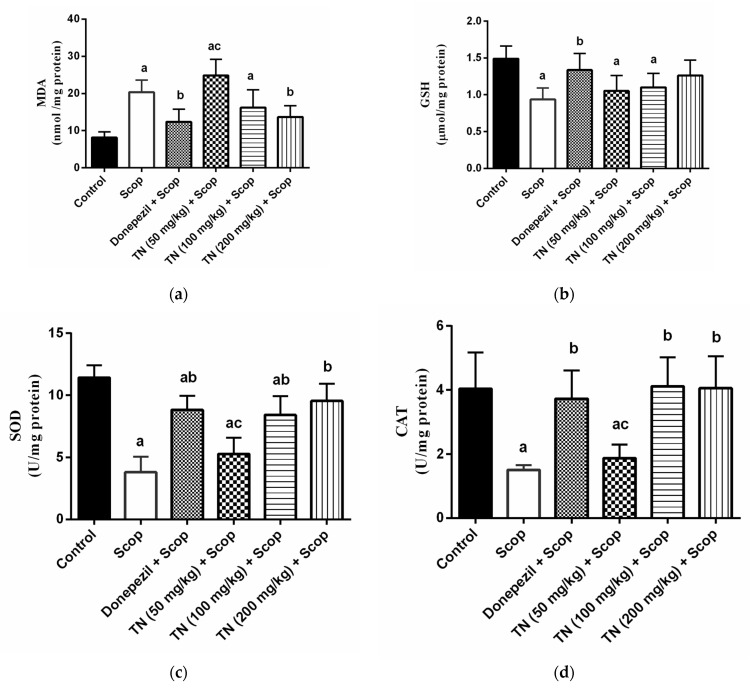
Effect of different doses of tiger nut on Scop-induced oxidative stress. (**a**) MDA, (**b**) GSH, (**c**) SOD and (**d**) CAT. Values are expressed as the mean ± SD (*n* = 6). Values are statistically significant at *p* < 0.05 versus the control group, *p* < 0.05 versus the Scop-treated group and *p* < 0.05 versus donepezil-treated group.

Scop produced a state of oxidative stress, revealed by the dramatic elevation in the hippocampal MDA level (by≈2.5-fold) along with a significant decline in hippocampal GSH content (by 37.03%) and SOD and CAT activity (by 66.65% and 62.61%, respectively) compared with the control animals. However, donepezil and TN 200 mg treatment ameliorated the Scop-induced increase in MDA levels (F (5, 30) = 16.71, *p* < 0.0001) by approximately 39.04% and 32.79% respectively. Interestingly, compared with the Scop group rats, only donepezil increased the GSH content (F (5, 30) = 6.481, *p* < 0.0003) by approximately 1.4-fold, while donepezil and TN 100 or 200 mg elevated the SOD activity (F (5, 30) = 28.82, *p* < 0.0001) by approximately 2.3-fold, 2.2-fold and 2.5-fold, respectively, as well as the CAT activity (F (5, 30) = 12.61, *p* < 0.0001) by approximately 2.5-fold, 2.7-fold and 2.7-fold, respectively (Figure 4a–d).

### 2.5. Effect of Different Doses of TN on Scop-Induced Neuroinflammation

**Figure 5:**
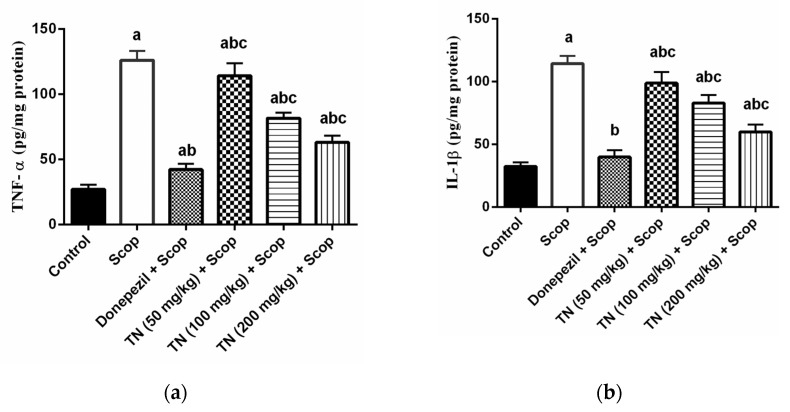
Effect of different doses of tiger nut on Scop-induced neuroinflammation. (**a**) TNF-α and (**b**) IL-1β. Values are expressed as the mean ± SD (*n* = 6). Values are statistically significant at *p* < 0.05 versus the control group, *p* < 0.05 versus the Scop-treated group and *p* < 0.05 versus the donepezil-treated group.

As shown in Figure 5a and b, Scop triggered inflammation via elevating the TNF-α and IL-1β levels by 4.7-fold and 3.5-fold, respectively, in comparison with the control rats. On the other hand, treatment with donepezil and TN 50 or 100 or 200 mg induced a marked improvement in Scop-induced neuroinflammation, as evidenced by the decrease in TNF-α levels (F (5, 30) = 252.3, *p* < 0.0001) by approximately 33.57%, 90.41%, 64.74% and 50.09%, respectively, and IL-1β levels (F (5, 30) = 165.6, *p* < 0.0001) by approximately 34.87%, 86.38%, 72.52% and 52.29%, respectively, of the Scop group values.

### 2.6. Effect of Different Doses of TN on Scop-Induced Apoptosis

**Figure 6:**
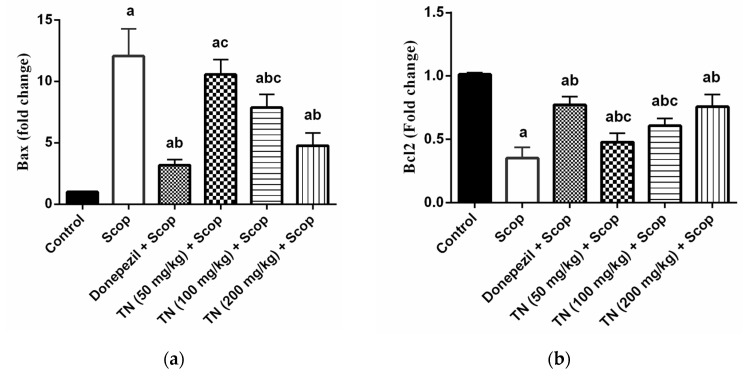

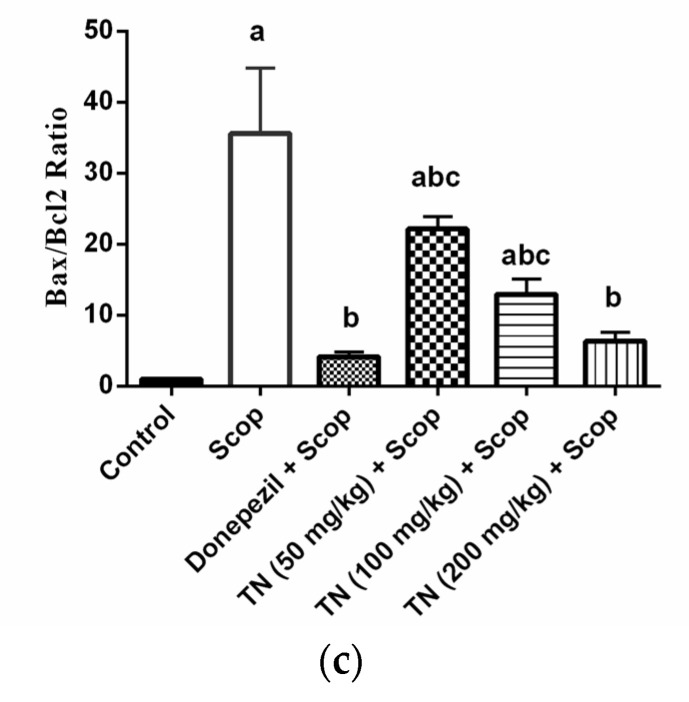
Effect of different doses of tiger nut on Scop-induced apoptosis. (**a**) Bax, (**b**) Bcl2 and (**c**) Bax/Bcl2 ratio. Values are expressed as the mean ± SD (*n* = 6). Values are statistically significant at *p* < 0.05 versus the control group, *p* < 0.05 versus the Scop-treated group and *p* < 0.05 versus donepezil-treated group.

Scop administration resulted in a dramatic up-regulation of the hippocampal pro-apoptotic Bax mRNA expression and the Bax/Bcl2 ratio by approximately 11.9-fold and 35.8-fold, respectively, along with a significant down-regulation of the hippocampal anti-apoptotic Bcl2 mRNA expression by approximately 34.72% compared with the control group. Donepezil and TN 100 or 200 mg significantly reversed the increase in the Bax mRNA expression (F (5, 30) = 77.24, *p* < 0.0001) by approximately 26.39%, 65.20% and 39.49%, respectively, in comparison with the Scop-treated rats. Additionally, treatment with donepezil and TN 50 or 100 or 200 mg attenuated Scop-induced apoptosis, as demonstrated by the increase in the Bcl2 mRNA expression (F (5, 30) = 68.25, *p* < 0.0001) by approximately 2.2-fold, 1.4-fold, 1.7-fold and 2.2-fold, respectively, as well as the reduction in the Bax/Bcl2 ratio (F (5, 30) = 65.76, *p* < 0.0001) by approximately 11.65%, 62.18%, 36.36% and 17.73%, respectively, of the Scop group values (Figure 6a–c).

### 2.7. Effect of Different Doses of TN on Scop-Induced Alterations in Aβ and β-Secretase Protein Expression

**Figure 7:**
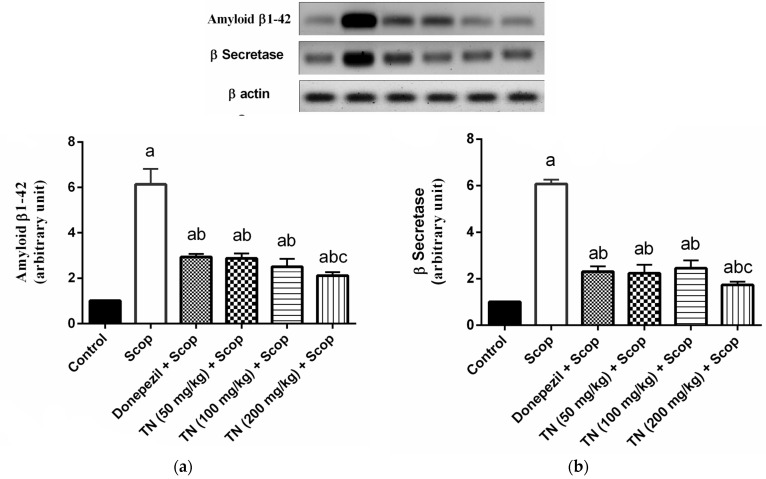
Effect of different doses of TN on Scop-induced alterations in (**a**) Aβ and (**b**) β-secretase protein expression. Values are expressed as the mean ± SD (*n* = 6). Values are statistically significant at *p* < 0.05 versus the control group, *p* < 0.05 versus the Scop-treated group and *p* < 0.05 versus donepezil-treated group. Representative western blots are depicted.

Scop markedly up-regulated the hippocampal Aβ and β-secretase protein expression by approximately 6-fold compared with the control animals. However, donepezil and TN 50 or 100 or 200 mg significantly ameliorated the increase in both the Aβ protein expression (F (5, 30) = 157.1, *p* < 0.0001) reaching approximately 47.79%, 46.88%, 40.74% and 34.54%, respectively, and the β-secretase protein expression (F (5, 30) = 313.5, *p* < 0.0001) reaching approximately 37.94%, 36.73%, 40.25% and 28.50%, respectively, in comparison with the Scop-treated rats. (Figure 7a,b).

### 2.8. Effect of Different Doses of TN on Scop-Induced Histopathological Alterations and Neuronal Loss

**Figure 8:**
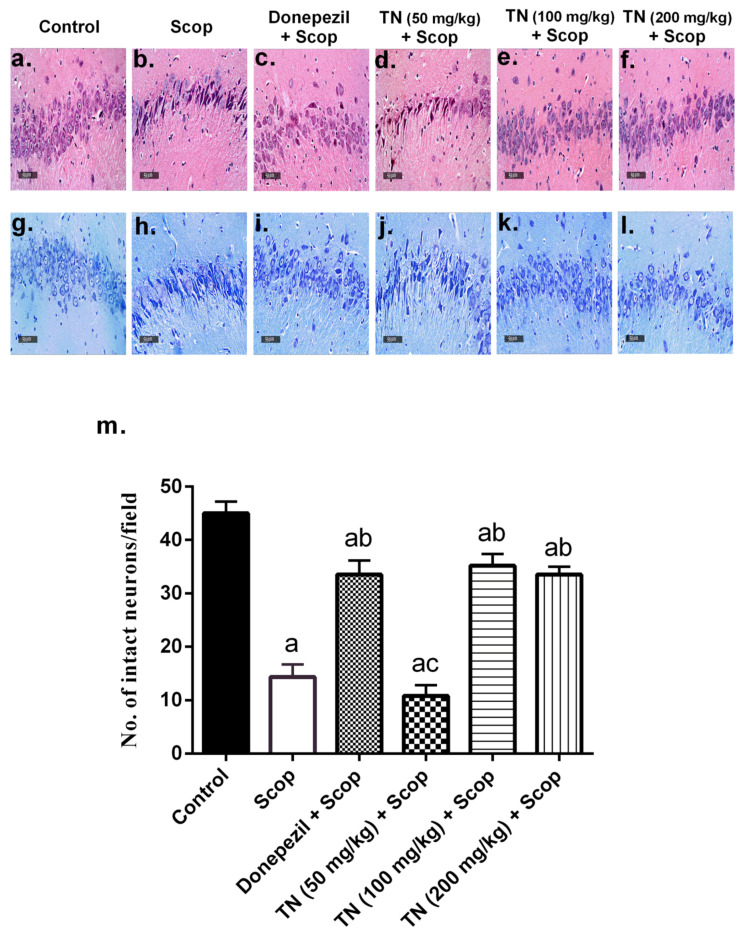
Effect of different doses of TN on Scop-induced histopathological alterations and neuronal loss. (**a**–**f**): specimens stained with H&E (400× magnification). (**a**) The control group showed a normal hippocampal structure; (**b**) the Scop group showed severe neuronal necrosis and extensive gliosis; (**c**) the donepezil group showed minimal neurodegenerative changes and persistent gliosis; (**d**) the TN 50 mg group showed severe neuronal necrosis and extensive gliosis; (**e**) the TN 100 mg group showed minimal neurodegenerative changes; (**f**) the TN 200 mg group showed minimal neurodegenerative changes and diminished gliosis. (**g**–**l**) Specimens stained with Nissl (400× magnification). (**m**) Number of Nissl-stained cells (intact neurons). Statistical analysis was carried out using one-way ANOVA followed by the Tukey–Kramer multiple comparison test. Values are expressed as the mean ± SD. Values are statistically significant at *p* < 0.05 versus the control group, *p* < 0.05 versus the Scop-treated group and *p* < 0.05 versus donepezil-treated group.

Photomicrographs from the control rats revealed no histopathological alterations, with normal histological features of the hippocampus showing intact pyramidal neurons and intracellular brain matrices (Figure 8a). Additionally, Nissl staining of the control group demonstrated normal intact neurons (Figure 8g,m). On the other hand, Scop-treated rats exhibited severe neuronal necrosis and degeneration, together with mild intracellular edema and extensive gliosis, compared with the control group (Figure 8b). Furthermore, marked neuronal loss was observed by Nissl staining (Figure 8h,m). The administration of donepezil produced significant neuroprotective effects, showing intact neurons with minimal neurodegenerative changes. However, persistent gliosis was observed (Figure 8c). Furthermore, donepezil ameliorated the neuronal loss, as evidenced by the Nissl staining (Figure 8i,m). Interestingly, TN 100 or 200 mg demonstrated marked neuroprotection, resembling donepezil showing intact neurons with minimal neurodegenerative changes, with TN 200 mg presenting diminished gliosis (Figure 8e,f). Furthermore, TN 100 or 200 mg amended the neuronal loss, verified by the Nissl staining (Figure 8k–m). However, TN 50 mg revealed negligible neuroprotective effects, exhibiting almost the same histological alterations seen in the Scop group (Figure 8d), with significant neuronal loss detected by Nissl staining (Figure 8j,m).

## 3. Discussion

TN extract offers antioxidant [44,45], anti-inflammatory, neuroprotective [46] and beneficial effects in memory-related disorders [47]. Therefore, the aim of the present study was to investigate the protective effect of TN extract on Scop-induced memory impairment in rats. Scop is a muscarinic cholinergic receptor antagonist, causing cognitive decline by increasing AChE activity, oxidative stress and neuroinflammation in the rat brain, thus developing AD-like symptoms [48,49]

The MWM is the most widely-employed behavioral test for studying hippocampal-spatial learning and reference memory in rodents. Moreover, it is used to recognize drugs capable of reducing or preventing memory loss, i.e., drugs with anti-amnesic properties [50]. Learning is defined as a decline over trials in the latency to locate the sunken platform. [51]. During the acquisition phase, the mean escape latency, which is the time each mouse spent to find the platform, was significantly increased in the Scop group, while treatment with donepezil significantly reversed this alteration. However, treatment with TN (100 and 200 mg/kg) significantly decreased the mean escape latency compared with the Scop group values. In the probe test, the time spent in the target quadrant was measured to indicate the animals’ ability to recall the precise location where the platform was previously retained [51]. The Scop-treated rats showed the least time spent in the target quadrant, indicating an impairment in spatial learning and memory. On the other hand, the administration of donepezil restored the time spent in the target quadrant to the control levels. TN extract (100 and 200 mg/Kg) presented the highest time spent in the target quadrant, indicating reestablished memory. The Y-maze is a spontaneous alteration behavioral test, based on the willingness of rodents to explore a completely new environment in order to understand their spatial learning and memory [52]. Alteration behavior is a measure of immediate spatial working memory, a form of short-term memory [53], and the number of arm entries serves as an indicator of locomotor activity [54]. In the present study, the Scop group demonstrated an increase in locomotor activity, as evidenced by a significant increase in the number of arm entries compared with the control group, in addition to a significant decrease in short-term memory performance, as demonstrated by the decreased spontaneous alteration percentage in relation to the control group. The administration of donepezil reversed the high locomotor activity and the low spontaneous alteration percentage. Treatment with TN (100 and 200 mg) showed a greater exploratory drive and that learning and short memory have been restored through the lowering of the locomotor activity and increasing the spontaneous alteration, thus ameliorating the decreased alteration behavior induced by Scop. Improvement in MWM and Y-maze measured parameters by treatment with TN (100 and 200 mg/kg) supported its beneficial effect in reestablishing the rats’ spatial learning, memory and exploratory behavior, which may indicate TN’s positive effects on postponing neurodegeneration. The fatty acid-enriched profile of TN extract could be associated with ameliorated spatial learning impairment, as tested by MWM. HE et al., [55] reported that maintaining high docosahexaenoic acid (DHA) levels in the brain, either endogenously or supplemented, significantly improved hippocampal neurogenesis, as represented by the higher number of proliferating neurons in addition to neuritogenesis. Additionally, the flavonoids also contribute to the neuroprotective effects of tiger nuts. For instance, luteolin, a major metabolite of TN, has been well-reported for its positive impact on the cognitive functions and spatial learning in an AD-induced-rat model [56]. Luteolin (at 10, 20 mg/kg) reduced the escape latency and the distance traveled in the Morris water maze, while the time spent in the target quadrant notably increased. Several studies have revealed that flavonoids such as luteolin exert this effect via the modulation of brain neurotransmitters acting on the cholinergic and glutamatergic systems [57].

It is well known that AD pathophysiology includes the formation of extracellular senile plaques, which consist of Aβ peptide aggregates [58], that are responsible for cognitive decline, memory loss and significant inflammatory response [59]. Excessive production of the neurotoxic Aβ peptide from the amyloid precursor protein (APP) cleavage is done by β-secretase, which is the rate-limiting enzyme in this process [60]. Therefore, the down-regulation of the β-secretase expression inhibits Aβ generation [61]. In our study, the Scop up-regulated the hippocampal Aβ and β-secretase expression compared with the control group. The administration of donepezil significantly lowered the Aβ1-42 and β-secretase expression, which correlates with the study of Patel and his colleagues [62]. Interestingly, the Aβ1-42- and β-secretase-lowering effects of TN at the doses of 50–200 mg were nearly comparable with those of the donepezil-treated group, which may suggest a beneficial effect of TN in decreasing the disease burden.

Furthermore, the accumulation of Aβ plaques can overstimulate microglia, which produce extensive amounts of pro-inflammatory cytokines (TNF-α and IL-1β), eliciting a cascade of neuroinflammation that mediate neurotoxicity and eventually AD [59]. In the present study, Scop induced a strong inflammatory response through the up-regulation of TNF-α and IL-1β levels in the hippocampus of rats. Treatment with donepezil alleviated the Scop-induced neuroinflammation by diminishing the TNF-α and IL-1β levels, signifying anti-inflammatory properties. These results correlate with previously demonstrated data [63,64]. TN (50, 100 and 200 mg/kg) treatment managed to mitigate Scop-induced neuroinflammation, suggesting the significant anti-inflammatory activity of TN, which was also reported for other *Cyperus* species [46] in a dose-dependent manner. The decrease in the pro-inflammatory cytokines may also prevent the over-activation of the hippocampal cells, thus can diminish Aβ accumulation [59]. This promising neuroprotective effect could be offered by the fatty acids as represented by linolenic acid, with a reported ameliorative effect on Aβ-induced glial-cell-mediated neuroinflammation and cognitive dysfunction in mice [65]. On the other hand, several mechanisms were postulated regarding the potential of flavonoids for decreasing Aβ accumulation [66]. This includes exerting an anti-amyloidogenic activity, interfering with the hyperphosphorylation of tau proteins and β-secretase inhibition [66]. Further, certain flavonoids, such as myricetin, quercetin, catechin and luteolin, are capable of modulating the signaling pathways implicated in neurodegeneration as represented by glycogen synthase kinase-3β (GSK-3β), phosphatidylinositol-3-kinase/ protein kinase B (PI3K/Akt), tyrosine kinase and the mitogen-activated protein kinase (MAPK) pathways [67,68,69].

Aβ peptide is documented to be associated with reactive oxygen species’ generation, leading to the aggregation of Aβ and plaque formation [70]. Oxidative stress elicits lipid peroxidation, together with decreased GSH and antioxidant enzymes, which leads to cholinergic neuronal damage and cognitive dysfunctions [71]. Oxidative stress is among the fundamental mechanisms of cell damage following the administration of Scop [72]. In our study, Scop produced a state of oxidative stress, revealed by the dramatic elevation in the hippocampal MDA level, a reliable oxidative stress marker, and the significant decline in hippocampal anti-oxidative defenses, which are GSH, SOD and CAT activities. Donepezil succeeded in decreasing hippocampal MDA and in increasing hippocampal GSH, SOD and CAT activity, indicating an increase in antioxidant defenses in the brain. These outcomes were apparently relevant with previous studies [73,74]. However, TN did not enhance antioxidant activity in all its measured parameters, where only TN (200 mg/kg) decreased the elevated hippocampal MDA, while TN (100 and 200 mg/kg) elevated the SOD and CAT activity only. This antioxidant effect is in part due to the flavonoid content. Several mechanisms of action were assigned for plant flavonoids, to include radical scavenging activity, enhancing the antioxidant enzymes while suppressing the oxidases in addition to metal chelation [75]. In the same context, Moghaddam et al., [76] reported the potential of hesperitin for increasing the antioxidant enzymes, as represented by SOD, CAT, glutathione peroxidase and glutathione reductase, thus resulting in the alleviation of the oxidative stress in the hippocampus.

In the present study, Scop-treated rats showed an increase in hippocampal AChE activity, leading to cognitive impairment and memory loss, as in tune with previously documented data [77], causing amplified acetylcholine degradation and impairment in learning and memory. Donepezil and its metabolites are reversible AChE inhibitors [78]. The administration of donepezil and TN (100 and 200 mg/kg) managed to increase cholinergic activity and to reverse the impairment of cognitive function through the inhibition of hippocampal AChE activity. These results suggest that TN could inhibit cholinergic neuronal loss and cognitive impairment. Long chain polyunsaturated fatty acids (LC-PUFA) constitute an integral part of the brain neuronal composition [79]. Alpha-linolenic acid (ALA, *n*-3) is converted in vivo to eicosapentaenoic acid (EPA) and DHA. Interestingly, the supplementation of DHA has been reported to improve cholinergic transmission in animal models [80]. Additionally, ALA has been recognized as a potential dietary AChE inhibitor [81]. On the other hand, several flavonoids, such as luteolin and naringenin, are able to provoke AChE inhibitory activity as reported both in animal models or in vitro studies [82,83]. This is quite relevant due to the presence of several flavonoids in the extract used, such as luteolin, cirsimaritin, apigenin, kaempferide, naringenin, kaempferol, isokaempferide and various derivatives of these. Flavonoids have the ability to inhibit the activity of cholinesterases, including AChE, butyrylcholinesterase and β-secretase, that are implicated in neuroprotective and cognitive functions. This family of phenolic compounds has shown that they can interact with several signaling protein pathways, such as PI3-kinase/Akt and ERK, and can modulate their actions, leading to biological benefits related to neuroprotection [27,28,83].

Previous persuasive data has proved that apoptotic mechanisms are a part of AD progression that are elicited by oxidative stress and inflammation. Thus, hindering both oxidative stress and inflammation and subsequently preventing apoptosis can account for diminishing neuronal damage and consequent cognitive impairment [84]. In the present study, Scop significantly up-regulated the hippocampal pro-apoptotic protein Bax mRNA level, which causes cell death [85], down-regulated the hippocampal anti-apoptotic Bcl2 mRNA level, which acts as an anti-apoptotic factor [86] and significantly up-regulated the Bax/Bcl2 ratio. Treatment with donepezil significantly reversed Scop-induced effects and managed to diminish the pro-apoptotic Bax mRNA level and the Bax/Bcl2 ratio and to increase the anti-apoptotic Bcl2 mRNA level. These results are consistent with the previous results [87,88]. The administration of TN extract (100 and 200 mg/kg) decreased the Bax mRNA overexpression, while all doses of TN extract (50, 100 and 200 mg/kg) promoted the Bcl2 mRNA expression, thus reducing the Bax/Bcl2 ratio, which indicates an anti-apoptotic effect.

Therefore, these results indicate that TN could ameliorate cognitive and memory dysfunction by diminishing Aβ aggregates, some oxidative stress and inflammation and subsequently modifying neural apoptosis. Similarly, previous reports have demonstrated the inhibitory effect of the total flavonoids of *Scutellaria baicalensis* on neuronal apoptosis, as elicited by amyloid beta-peptide. This effect was evoked by the decreased expression of the pro-apoptotic protein Bax, cytochrome c and caspase-3, concurrent with the increased expression level of Bcl2, in a dose-dependent matter [89]. Regarding the lipid profile, n-3 fatty acids are well documented to maintain a healthy nervous system [90]. In a previous study by Ajami et al., [91], long-term administration (21 days) of a mixture of DHA and EPA supplements before inducing ischemia in the hippocampus of rats, increased the Bcl-2 expression level and decreased the Bax expression 48 h after ischemia, together with a reduced count of neuronal cell loss in the hippocampus.

The histopathological examination of sections from the Scop group revealed neurodegeneration, with extensive gliosis and neuronal loss, while treatment with donepezil showed minimal neurodegeneration and neuronal loss, with less gliosis. Sections from the TN (100 and 200 mg/kg) groups demonstrated marked neuroprotection, as evidenced by intact neurons with minimal neurodegeneration. However, diminished gliosis was obvious with the TN (200 mg/kg) extract.

## 4. Materials and Methods

### 4.1. Plant Material and Extraction

*Cyperus esculentus* L. rhizomes were purchased from a local market “Harraz”, Cairo, Egypt. The identity of the plant was confirmed by staff members at the Egyptian Agricultural Museum. A voucher specimen (20.5.2020.1) was deposited at the herbarium of Pharmacognosy Department, Faculty of Pharmacy, Cairo University, Cairo, Egypt. One Kg of tiger nut rhizomes were extracted by maceration, using ethanol (analytical grade, El-Gomhuria Chemical Company, Cairo, Egypt), being an inexpensive and simple conventional method for the extraction of plant material [92]. The filtered extract was then evaporated using a rotary evaporator and the resultant oily extract was kept in a refrigerator at −8 °C for further analysis.

### 4.2. UHPLC-ESI-QTOF-MS Profiling

A solution at a concentration of 10 mg/mL was prepared from the dry extracts for analysis by mass-spectrometry coupled with liquid-chromatography. Specifically, samples were analysed using an ACQUITY UPLC H-Class System (Waters, Milford, MA, USA) coupled with a QTOF-MS (Synapt G2, Waters Corp., Milford, MA, USA). The chemical compounds were separated using a reversed-phase C18 analytical column (Agilent Zorbax Eclipse Plus, 1.8 μm, 4.6 × 150 mm) at 22 °C. The mobile phases were H_2_O containing 0.5% of acetic acid and methanol as solvent A and B, respectively. The following mobile phase gradient was used in order to achieve an efficient separation: 0.0 min (A:B 100/0), 15.0 min (A:B 40/60), 33.0 min (A:B 0/100), 46.0 min (A:B 0/100) and 55.0 min (A:B 100/0). The flow rate and the injection volume were 400 µL/min and 10 µL, respectively. Detection was performed in an electrospray negative-ion mode (ESI-) over a range from 50 to 1200 *m*/*z*. The MS acquisition was performed using two parallel scan functions by rapid switching, in which one scan was operated at a low collision energy in the gas cell (4 eV) and the other at an elevated collision energy (MS^E^ energy linear ramp: from 20 to 60 eV). Leucine enkephalin was injected continuously during the analysis for mass calibration at a concentration of 300 ng/mL. Other MS parameters were as follows: capillary voltage 2.2 kV, cone voltage 30 V; desolvation temperature 500 °C; desolvation gas flow 700 L/h; cone gas flow 50 L/H; source temperature 100 °C; scan duration 0.1 s, resolution 20000 FWHM.

### 4.3. UHPLC-ESI-QTOF-MS Data Processing

Firstly, the raw data files were transformed to an mzML format using MSConverGUI software [93]. The MS data were processed through the open-source software MZmine 2.53 [94,95]. A noise level of 1.0 × 10^3^ was selected. An ADAP chromatogram builder method was used under the following parameters: MS level: 1; min number of scans: 9; group intensity threshold: 1.0 × 10^3^; min highest intensity: 1.0 × 10^4^; *m*/*z* tolerance: 10 ppm. After that, the chromatogram was deconvoluted and was performed using the wavelets (ADAP) algorithm and the following parameters: S/N threshold: 50; min feature height: 5 × 10^4^; coefficient/area threshold: 110; peak duration range: 0.05–0.3 min; RT wavelet range: 0–0.30. An isotopic peak grouper algorithm was also applied (*m*/*z* tolerance: 10 ppm; RT tolerance: 0.02 min, maximum charge: 2). The obtained features were aligned between samples using the “Join Aligner” algorithm, an *m*/*z* tolerance of 10 ppm and a RT tolerance of 0.1 min. The molecular features, which were also detected in blank samples, were removed from the final dataset. Finally, the molecular formulas of the final features were predicted using Sirius 4.4.29 [96], and the biological identities were annotated by comparing the MS/MS spectra of different databases (e.g., MoNA, Massbank, HMDB, FoodDB, etc.) with the fragments detected in the MS^E^ scans.

### 4.4. Biological Study

#### 4.4.1. Animals

Adult male Wistar rats (4 months old) weighing 150–200 g were provided by the animal facility of the Faculty of Pharmacy Cairo University, Egypt and were housed under controlled environmental conditions of constant temperature (22 ± 2 °C), relative humidity of 60 ± 10%, and a light/dark cycle (12/12-h). The rats were fed with standard chow diet and water was provided ad libitum. The experimental protocol was approved by the Ethics Committee for Animal Experimentation (PT: 3081) and adheres strictly to the recommendations of the National Institutes of Health Guide for Care and Use of Laboratory Animals (2011).

#### 4.4.2. Drugs and Chemicals

Scop hydrobromide trihydrate and tween 80 were purchased from Sigma-Aldrich Co. (St Louis, MO, USA). Donepezil was purchased from Pfizer Pharmaceuticals Company (Cairo, Egypt). Scop was dissolved in a saline solution (0.9% NaCl) and injected intraperitoneally (i.p.) at a volume of 1 mL/kg. Donepezil was dissolved in saline and administrated orally (p.o.) at a volume of 5 mL/kg. All other chemicals were of the highest analytical grade.

#### 4.4.3. Experimental Design

As depicted in Figure 2a, the rats were acclimatized for 1 week and randomly divided into six groups, each containing 15 animals. The whole experimental schedule was followed for 14 consecutive days. Group I: rats received saline i.p. and 1% tween 80 p.o. for 14 days and served as the control group. Group II: rats received 1% tween 80 p.o. for 14 days and Scop (1 mg/kg, i.p.) 30 min before the behavioral experiments on all days of behavioral testing [97]. Group III: rats received donepezil (5 mg/kg, p.o.) dissolved in saline for 14 days [98] and Scop as group II and served as the standard drug group. Group IV: rats received TN extract (50 mg/kg, p.o.) suspended in 1% tween 80 for 14 days and Scop as group II. Group V: rats received TN extract (100 mg/kg, p.o.) for 14 days and Scop as group II. Group VI: rats received TN extract (200 mg/kg, p.o.) for 14 days and Scop as group II. Scop was administrated 1 h after vehicle or treatment administration and 30 min before the behavioral experiments on all days of behavioral testing. On the last day of injection (day 14), neurobehavioral tests were carried out, including the Morris water maze (MWM) and the Y-maze tests.

#### 4.4.4. Behavioral Assessments

##### Morris Water Maze Test

The MWM test is used to evaluate spatial learning and memory in animal models [99,100]. The maze consisted of a stainless-steel circular pool (210 cm in diameter, 51 cm high) divided into four equal quadrants and filled with water (26 ± 2 °C) to a depth of 35 cm. A black hidden escape platform was placed inside the target quadrant, 2 cm below the water surface. The platform was kept at a fixed position during the time of training. A non-toxic dye was added to make the water opaque so that the platform was made invisible. Memory acquisition trials (120 s/trial) were performed two times a day for four consecutive days, with an interval of at least 15 min between the trials. During each acquisition trial, animals were left free to explore the pool and to search for the hidden platform. Once the rat located the platform, it was left there for an additional 20 s to rest, while if an animal failed to reach the platform within 120 s it was gently guided to it and kept there for 20 s. The mean escape latency was calculated as the time taken by each rat to locate the hidden platform and was used as an index of acquisition or learning. On the fifth day, the rats were subjected to a probe-trial session where the platform was removed from the pool and each rat was allowed to explore the pool for 60 s. The time spent by each rat in the target quadrant in which the hidden platform was previously placed was taken as an index of retrieval or memory.

##### Y-Maze Test

The Y-maze test is used to measure the spatial working memory in rodents [101]. The maze was composed of 3 identical arms, 40 cm long, 35 cm high and 12 cm wide, positioned at equal angles (labeled A, B and C). The rats were placed in the center of the Y-maze, facing the south arm B, and were allowed to move freely through the maze for a period of 5 min. Spontaneous alternation was examined by visually recording the pattern of entrance into each arm in the maze for each rat. Arm entry was scored when the hind paws of the rat were completely placed in the arm. Consecutive entry into the three arms on an overlapping triplet set was defined as spontaneous alternation, i.e., BCA, ABC or CAB. Accordingly, the alternation percentage was calculated as the number of spontaneous alterations × 100 / total number of entries.

#### 4.4.5. Brain Processing

Twenty-four hours after the end of the behavioral testing, rats were euthanized by cervical dislocation under light anesthesia and brains were rapidly dissected, washed with ice-cold saline and divided into three sets. In the first set (*n* = 3), the brains were fixed in 10% (*v*/*v*) formalin for 24 h to perform histopathological staining. In the other sets, the hippocampi were rapidly dissected and stored at −80 °C. The hippocampi from the rats in the second set (*n* = 6) were homogenized in ice-cold physiological saline to prepare a 10% homogenate and used for ELISA and colorimetric assay. The hippocampi from the rats in the third set (*n* = 6) were used for real-time PCR and Western blot analyses.

#### 4.4.6. Biochemical Measurements

##### Acetylcholinesterase Activity

According to the manufacturer’s instructions, the hippocampal level of AChE activity was determined using an AChE assay kit (Abcam, Cambridge, UK). The AChE activity assay protocol uses 5,5-dithiobis 2-nitrobenzoic acid (DTNB) to quantify the thiocholine produced from the degradation of acetylthiocholine iodide by AChE. The absorption intensity of the DTNB adduct (412 nm) is proportional to the AChE activity. The results are expressed as U/mg protein.

##### Determination of Oxidative Stress Biomarkers

Malondialdehyde (MDA) was measured in the hippocampal homogenate by determining the thiobarbituric acid reactive substances, according to the method described by [102]. Moreover, the hippocampal glutathione (GSH) content was determined using Ellman’s reagent, according to the method described by [103]. The results are expressed as nmol/mg protein and µmol/mg protein, respectively.

The activity of superoxide dismutase (SOD) and catalase (CAT) were measured colorimetrically in the hippocampal homogenate using commercially available kits (Bio-diagnostic kit, Giza, Egypt) as instructed by the manufacturer. The results are expressed as U/mg protein.

##### Enzyme-Linked Immunosorbent Assay

Hippocampal TNF-α and IL-1β levels were estimated using rat ELISA kits purchased from R&D Systems Inc. (Minneapolis, MN, USA). The procedures were performed according to the manufacturer’s instructions. The results are expressed as pg/mg protein.

##### Quantitative Real-Time Polymerase Chain Reaction

Total RNA was extracted from hippocampal tissues using an RNeasy Kit (Qiagen, Valencia, CA, USA) and the purity of the obtained RNA was verified spectrophotometrically by recording the optical density at 260/280 nm. Equal amounts of RNA were then reverse transcribed into cDNAs using an RT-PCR kit (Fermentas, Waltham, MA, USA) according to the manufacturer’s guidelines. Quantitative RT-PCR was performed to assess the expression of the Bax and Bcl2 mRNAs using a SYBR Green PCR Master Mix (Applied Biosystems, Foster City, CA) according to the manufacturer’s instructions. Briefly, 1 μg of total RNA was mixed with 50 μM oligo (dT) 20, 50 ng/μL random primers and 10 mM dNTP mix in a total volume of 10 μL. The primer sequences used in the present study are: Bax forward 5ʹCTGCAGAGGATGATTGCTGA3ʹ, Bax reverse: 5ʹCATCAGCTCGGGCACCTTTAG3ʹ, Bcl-2 forward 5ʹGCTACGAGTGGGATACTGG3ʹ, Bcl-2 reverse 5ʹGTGTGCAGATGCCGTTCA3ʹ and β-actin forward 5′CGTTGACATCCGTAAAGACCTC3′ and β-actin 5′reverse TAGGAGCCAGGGCAGTAATCT3′. The thermal cycler protocol consisted of an initial enzyme activation step at 95 °C for 5 min, followed by 40 cycles of 5 s of denaturation at 95 °C and 10 s of annealing/extension at 60 °C. The relative expression of the target gene was obtained using the 2^−ΔΔCT^ formula. All values were normalized to β-actin levels and presented as fold changes.

##### Western Blot Analysis

Hippocampal tissues were homogenized in a lysis buffer and the protein content was measured using a Bradford assay kit (Bio-Rad, USA). Briefly, equal amounts of protein (20 μg) were separated by SDS-PAGE and transferred to polyvinylidene difluoride membranes (Pierce, Rockford, IL) using a Bio-Rad Trans-Blot system. The membranes were blocked with a blocking solution composed of 20 mM Tris-Cl (pH 7.5), 150 mM NaCl, 0.1% Tween 20 and 3% bovine serum albumin and incubated overnight at 4 °C with one of the following primary antibodies (1:1000): Aβ (1–42), β-secretase 1 or β-actin obtained from Thermo Fisher Scientific Inc. (Rockford, IL). The filters were washed and subsequently probed with peroxidase-labeled secondary antibodies. Finally, the band intensity was analyzed using a ChemiDoc imaging system with Image LabTM software version 5.1 (Bio-Rad Laboratories Inc., Hercules, CA, USA). The results were presented as arbitrary units after normalization to levels of the β-actin protein expression.

#### 4.4.7. Histopathological Examination

The brains were carefully removed, rinsed with ice-cold saline and immediately fixed with 10% neutral buffered formalin for 72 h. Samples were processed and dehydrated in serial grades of ethanol, cleared in xylene, then infiltrated and embedded into Paraplast plus tissue embedding media. Coronal brain sections were processed for paraffin embedding and 4 μm sections were cut by a rotatory microtome and mounted on glass slides. Sections were then stained with hematoxylin and eosin (H&E) and examined under a light microscope. Nissl staining was also performed to demonstrate degenerated and intact neurons in the hippocampus. Sections were stained with Cresyl violet dye (1% *w*/*v* in water) for 5 min, air dried at room temperature for 1 h and then briefly immersed in alcohol. The average number of intact neurons was quantified from six random non-overlapping fields in the hippocampus in Nissl-stained tissue sections for each sample. All morphological examinations, photographs as well as quantitative analysis were recorded using a Full HD microscopic camera operated by Leica Microsystems (GmbH, Wetzlar, Germany).

#### 4.4.8. Statistical Analysis

The data are presented as the mean ± S.D. Data were analyzed using one-way ANOVA followed by the Tukey–Kramer multiple comparison test. GraphPad Prism software (version 7.04; GraphPad Software, Inc., San Diego, CA, USA) was used to perform the statistical analysis and to present the data. The level of significance was fixed at *p* < 0.05 for all statistical tests.

## 5. Conclusions

The current study discusses the detailed metabolic profiling of *Cyperus esculentus,* which resulted in the putative annotation of 88 metabolites including saccharides, amino acids, organic acids, fatty acids, phenolic compounds and flavonoids. In conclusion, it reveals that the TN extract can significantly attenuate Scop-induced memory impairments by diminishing Aβ aggregates, as well as its anti-inflammatory, antioxidant, anti-apoptotic and anti-AChE activities. Therefore, TN may have immense therapeutic and prophylactic potential for the treatment of neurodegenerative cognitive impairment. The presence of polyphenols, especially flavonoids, as well as fatty acids in the TN extract could be correlated with the observed bioactive effects. Nevertheless, future studies are needed to isolate the active ingredient(s) and to reveal the corresponding potential mechanism of action.

## Abbreviations

Aβ.amyloid-betaADAlzheimer’s diseaseAChEacetylcholinestraseALAalpha-linolenic acidBaxBcl2-associated X proteinBcl-2B-cell lymphoma 2CATcatalaseDHAdocosahexaenoic acidDTNB5,5-dithiobis 2-nitrobenzoic acidEPAeicosapentaenoic acidGSHglutathioneGSK-3βglycogen synthase kinase-3βIL-1βinterleukin 1 betaLC-PUFAlong chain polyunsaturated fatty acidsMAPKmitogen-activated protein kinaseMDAmalodialdehydeMWMMorris water mazePI3K/AKTphosphatidylinositol-3-kinase/protein kinase BScopscopolamineSODsuperoxide dismutaseTNtiger nutTNF-αtumor necrosis factor alphaUHPLC-ESI-QTOF-MSultra performance liquid chromatography with electrospray ionization and quadrupole time-of-flight mass spectrometry

## Data Availability

Not Applicable.

## References

[1] Fawzi S.F., Menze E.T., Tadros M.G. (2020). Deferiprone ameliorates memory impairment in Scopolamine-treated rats: The impact of its iron-chelating effect on beta-amyloid disposition. Behav. Brain. Res..

[2] Thakur A.K., Kamboj P., Goswami K., Ahuja K.J.J.A.P.R. (2018). Pathophysiology and management of Alzheimer’s disease: An overview. J. Anal. Pharm. Res.

[3] Tang K.S. (2019). The cellular and molecular processes associated with scopolamine-induced memory deficit: A model of Alzheimer’s biomarkers. Life Sci..

[4] Aisen P.S., Cummings J., Jack C.R., Morris J.C., Sperling R., Frölich L., Jones R.W., Dowsett S.A., Matthews B.R., Raskin J.J.A.s.r. (2017). On the path to 2025: Understanding the Alzheimer’s disease continuum. Alzheimer’s Res. Ther..

[5] Tiwari S., Soni R.J.J.A.D.P. (2014). Alzheimer’s disease pathology and oxidative stress: Possible therapeutic options. J. Alzheimers. Dis Park..

[6] Shabani S., Mirshekar M.A. (2018). Diosmin is neuroprotective in a rat model of scopolamine-induced cognitive impairment. Biomed. Pharmacother..

[7] Fan Y., Hu J., Li J., Yang Z., Xin X., Wang J., Ding J., Geng M. (2005). Effect of acidic oligosaccharide sugar chain on scopolamine-induced memory impairment in rats and its related mechanisms. Neurosci. Lett..

[8] Kumar A., Singh A., Ekavali (2015). A review on Alzheimer’s disease pathophysiology and its management: An update. Pharmacol. Rep..

[9] Gambo A., Da’u A. (2014). Tiger nut (*Cyperus esculentus*): Composition, products, uses and health benefits-a review. Bayero. J. Pure. Appl. Sci..

[10] Oderinde R., Tairu O. (1988). Evaluation of the properties of yellow nutsedge (*Cyperus esculentus*) tuber oil. Food Chem..

[11] Ejoh R.A., Djomdi, Ndjouenkeu R. (2006). Characteristics of tigernut (*Cyperus esculentus*) tubers and their performance in the production of a milky drink. J. Food Process. Preserv..

[12] Tackholm V. (1974). Students’ flora of Egypt.

[13] Obadina A., Oyawole O., Ayoola A., VC B. (2008). Quality assessment of gari produced using rotary drier. Food Processing: Methods, Techniques and Trends.

[14] Arafat S.M., Gaafar A.M., Basuny A.M., Nassef S.L. (2009). Chufa tubers (*Cyperus esculentus* L.): As a new source of food. World Appl. Sci. J..

[15] Yu Y., Lu X., Zhang T., Zhao C., Guan S., Pu Y., Gao F. (2022). Tiger Nut (*Cyperus esculentus* L.): Nutrition, Processing, Function and Applications. Foods.

[16] Yeboah S.O., Mitei Y.C., Ngila J.C., Wessjohann L., Schmidt J. (2012). Compositional and structural studies of the oils from two edible seeds: Tiger nut, *Cyperus esculentum*, and asiato, Pachira insignis, from Ghana. Food Res. Int..

[17] Nofouzi K., Mahmudi R., Tahapour K., Amini E., Yousefi K. (2016). *Verbascum speciosum* methanolic extract: Phytochemical components and antibacterial properties. J. Essent. Oil Bear Plants.

[18] Allahyari S., Pakbin B., Amani Z., Mahmoudi R., Hamidiyan G., Peymani A., Qajarbeygi P., Mousavi S. (2021). Antiviral activity of *Phoenix dactylifera* extracts against herpes simplex virus type 1: An animal study. Comp. Clin. Pathol..

[19] Nwosu L.C., Edo G.I., Ozgor E. (2022). The phytochemical, proximate, pharmacological, GC-MS analysis of *Cyperus esculentus* (Tiger nut): A fully validated approach in health, food and nutrition. Food Biosci..

[20] Sánchez-Zapata E., Fernández-López J., Angel Pérez-Alvarez J. (2012). Tiger nut (*Cyperus esculentus*) commercialization: Health aspects, composition, properties, and food applications. Compr. Rev. Food Sci. Food Saf..

[21] Abimbade S.F., Oloyede G.K., Nwabueze C.C. (2014). Antioxidant and toxicity screenings of extracts obtained from *Cyperus esculentus*. Acad. Arena.

[22] Saber F.R., Mahrous E.A., Ferranti P. Novel Functional Foods From Plants of the Mediterranean Area: Biological, Chemical, Metabolomic Approaches. Reference Module in Food Science.

[23] Sumner L.W., Amberg A., Barrett D., Beale M.H., Beger R., Daykin C.A., Fan T.W.-M., Fiehn O., Goodacre R., Griffin J.L. (2007). Proposed minimum reporting standards for chemical analysis. Metabolomics.

[24] Ijarotimi O.S., Yinusa M.A., Adegbembo P.A., Adeniyi M.D. (2018). Chemical compositions, functional properties, antioxidative activities, and glycaemic indices of raw and fermented tigernut tubers (Cyperus esculentus Lativum) flour. J. Food Biochem..

[25] Bazinet R.P., Layé S. (2014). Polyunsaturated fatty acids and their metabolites in brain function and disease. Nat. Rev. Neurosci..

[26] Song J., Kim Y.-S., Lee D.H., Lee S.H., Park H.J., Lee D., Kim H. (2019). Neuroprotective effects of oleic acid in rodent models of cerebral ischaemia. Sci. Rep..

[27] Ayaz M., Sadiq A., Junaid M., Ullah F., Ovais M., Ullah I., Ahmed J., Shahid M. (2019). Flavonoids as prospective neuroprotectants and their therapeutic propensity in aging associated neurological disorders. Front. Aging. Neurosci..

[28] Kempuraj D., Thangavel R., Kempuraj D.D., Ahmed M.E., Selvakumar G.P., Raikwar S.P., Zaheer S.A., Iyer S.S., Govindarajan R., Chandrasekaran P.N. (2021). Neuroprotective effects of flavone luteolin in neuroinflammation and neurotrauma. Biofactors.

[29] Szwajgier D., Borowiec K., Pustelniak K. (2017). The neuroprotective effects of phenolic acids: Molecular mechanism of action. Nutrients.

[30] Marim F.M., Teixeira D.C., Queiroz-Junior C.M., Valiate B.V.S., Alves-Filho J.C., Cunha T.M., Dantzer R., Teixeira M.M., Teixeira A.L., Costa V.V. (2021). Inhibition of Tryptophan Catabolism Is Associated With Neuroprotection During Zika Virus Infection. Front. Immunol..

[31] Wang J., Song Y., Gao M., Bai X., Chen Z. (2016). Neuroprotective effect of several phytochemicals and its potential application in the prevention of neurodegenerative diseases. Geriatrics.

[32] Aljuhaimi F., Şimşek Ş., Özcan M.M. (2018). Comparison of chemical properties of taro (Colocasia esculenta L.) and tigernut (*Cyperus esculentus*) tuber and oils. J. Food Process Preserv..

[33] Bosch L., Alegria A., Farre R. (2005). RP-HPLC determination of tiger nut and orgeat amino acid contents. Food Sci. Technol. Int..

[34] Soto Mayer L. (2019). Phytochemical Analysis of the methanolic extract of tigernut, tuber of *Cyperus esculentus*, by ultra-high performance liquid chromatography coupled with electrospray ionization-quadrupole-time of flight-mass spectrometry (UHPLC/ESI-Q.-TOF-MS). M.Sc. Thesis.

[35] Abd-ElGawad A.M., Elshamy A.I., Al-Rowaily S.L., El-Amier Y.A. (2019). Habitat Affects the Chemical Profile, Allelopathy, and Antioxidant Properties of Essential Oils and Phenolic Enriched Extracts of the Invasive Plant *Heliotropium Curassavicum*. Plants.

[36] Elshamy A.I., Farrag A.R.H., Ayoub I.M., Mahdy K.A., Taher R.F., Gendy A.E.-N.G., Mohamed T.A., Al-Rejaie S.S., Ei-Amier Y.A., Abd-EIGawad A.M. (2020). UPLC-qTOF-MS phytochemical profile and antiulcer potential of *Cyperus conglomeratus* Rottb. alcoholic extract. Molecules.

[37] Sayed H.M., Mohamed M.H., Farag S.F., Mohamed G.A., Omobuwajo O.R., Proksch P. (2008). Fructose-amino acid conjugate and other constituents from *Cyperus rotundus* L.. Nat. Prod. Res..

[38] Sayed H.M., Mohamed M.H., Farag S.F., Mohamed G.A. (2001). Phytochemical and biological investigations of *Cyperus rotundus* L.. Bull. Facul.t Pharm. Cairo. Uni..

[39] Rocha F.G., de Mello Brandenburg M., Pawloski P.L., da Silva Soley B., Costa S.C.A., Meinerz C.C., Baretta I.P., Otuki M.F., Cabrini D.A. (2020). Preclinical study of the topical anti-inflammatory activity of *Cyperus rotundus* L. extract (Cyperaceae) in models of skin inflammation. J. Ethnopharmacol..

[40] El-Habashy I., Mansour R., Zahran M., El-Hadidi M., Saleh N. (1989). Leaf flavonoids of *Cyperus* species in Egypt. Biochem. Syst. Ecol..

[41] Uysal S., Zengin G., Sinan K.I., Ak G., Ceylan R., Mahomoodally M.F., Uysal A., Sadeer N.B., Jekő J., Cziáky Z. (2021). Chemical characterization, cytotoxic, antioxidant, antimicrobial, and enzyme inhibitory effects of different extracts from one sage (Salvia ceratophylla L.) from Turkey: Open a new window on industrial purposes. RSC Adv..

[42] Allan R., Wells R., MacLeod J. (1973). Flavanone quinones from *Cyperus* species. Tetrahedron Lett..

[43] Farrag A.R.H., Abdallah H.M., Khattab A.R., Elshamy A.I., El Gendy A.E.-N.G., Mohamed T.A., Farag M.A., Efferth T., Hegazy M.-E.F. (2019). Antiulcer activity of *Cyperus alternifolius* in relation to its UPLC-MS metabolite fingerprint: A mechanistic study. Phytomedicine.

[44] Innih S.O., Eluehike N., Francis B. (2021). Effects of aqueous extract of *Cyperus esculentus* (tiger nut) on antioxidant status and hematological indices in the heart of cadmium-induced wistar rats. Niger. J. Experiment. Clin. Biosci..

[45] Sudha T.S. (2021). Evaluation of anticonvulsant and antioxidant properties of *Cyperus esculentus* Linn. in various types of experimentally induced seizures in rats. Int. J. Green Pharm..

[46] Hussein J.S., Medhat D., Abdel-Latif Y., Morsy S., Gaafar A.A., Ibrahim E.A., Al-kashef A.S., Nooman M.U. (2021). Amelioration of neurotoxicity induced by esfenvalerate: Impact of *Cyperus rotundus* L. tuber extract. Comparat Clin. Pathol..

[47] Umukoro S., Okoh L., Igweze S.C., Ajayi A.M., Ben-Azu B. (2020). Protective effect of *Cyperus esculentus* (tiger nut) extract against scopolamine-induced memory loss and oxidative stress in mouse brain. Drug Metab. Person. Ther..

[48] El-Marasy S.A., Abd-Elsalam R.M., Ahmed-Farid O.A. (2018). Ameliorative effect of silymarin on scopolamine-induced dementia in rats. Maced. Journal Med. Sci..

[49] Barai P., Raval N., Acharya S., Borisa A., Bhatt H., Acharya N. (2019). Neuroprotective effects of bergenin in Alzheimer’s disease: Investigation through molecular docking, in vitro and in vivo studies. Behav. Brain. Res..

[50] Kim M.-S., Lee D.Y., Lee J., Kim H.W., Sung S.H., Han J.-S., Jeon W.K. (2018). *Terminalia chebula* extract prevents scopolamine-induced amnesia via cholinergic modulation and anti-oxidative effects in mice. BMC Complem Altern. Med..

[51] Tucker L.B., Velosky A.G., McCabe J.T. (2018). Applications of the Morris water maze in translational traumatic brain injury research. Neurosci. Biobehav. Rev..

[52] Birla H., Keswani C., Rai S.N., Singh S.S., Zahra W., Dilnashin H., Rathore A.S., Singh S.P. (2019). Neuroprotective effects of Withania somnifera in BPA induced-cognitive dysfunction and oxidative stress in mice. Behav. Brain Funct..

[53] Sarter M., Bodewitz G., Stephens D.N. (1988). Attenuation of scopolamine-induced impairment of spontaneous alternation behaviour by antagonist but not inverse agonist and agonist β-carbolines. Psychopharmacology.

[54] Brinza I., Boiangiu R.S., Hancianu M., Cioanca O., Erdogan Orhan I., Hritcu L. (2021). Bay Leaf (*Laurus Nobilis,* L.) Incense Improved Scopolamine-Induced Amnesic Rats by Restoring Cholinergic Dysfunction and Brain Antioxidant Status. Antioxidants.

[55] He C., Qu X., Cui L., Wang J., Kang J.X. (2009). Improved spatial learning performance of fat-1 mice is associated with enhanced neurogenesis and neuritogenesis by docosahexaenoic acid. Proc. Natl. Acad. Sci. USA.

[56] Wang H., Wang H., Cheng H., Che Z. (2016). Ameliorating effect of luteolin on memory impairment in an Alzheimer’s disease model. Mol. Med. Rep..

[57] Bakoyiannis I., Daskalopoulou A., Pergialiotis V., Perrea D. (2019). Phytochemicals and cognitive health: Are flavonoids doing the trick?. Biomed. Pharmacother..

[58] Selkoe D.J., Hardy J. (2016). The amyloid hypothesis of Alzheimer’s disease at 25 years. EMBO Mol. Med..

[59] Wang W.-Y., Tan M.-S., Yu J.-T., Tan L. (2015). Role of pro-inflammatory cytokines released from microglia in Alzheimer’s disease. Annal. Transl. Med..

[60] Das H., Sarkar S., Paidi R.K., Biswas S.C. (2021). Subtle genomic DNA damage induces intraneuronal production of amyloid-β (1-42) by increasing β-secretase activity. FASEB J..

[61] Fourriere L., Gleeson P.A. (2021). Amyloid β production along the neuronal secretory pathway: Dangerous liaisons in the Golgi?. Traffic.

[62] Patel P., Shah J.S. (2021). Effect of Vitamin D Supplementation on the Progression of Alzheimer’s Disease in Rats: A Mechanistic Approach. Res. Sq. Prepr..

[63] Djeuzong E., Kandeda A.K., Djiogue S., Stéphanie L., Nguedia D., Ngueguim F., Djientcheu J.P., Kouamouo J., Dimo T. (2021). Antiamnesic and Neuroprotective Effects of an Aqueous Extract of *Ziziphus jujuba* Mill.(Rhamnaceae) on Scopolamine-Induced Cognitive Impairments in Rats. Evid-Based Compl. Alt. Med..

[64] Kandeda A.K., Nguedia D., Ayissi E.R., Kouamouo J., Dimo T. (2021). *Ziziphus jujuba* (Rhamnaceae) Alleviates Working Memory Impairment and Restores Neurochemical Alterations in the Prefrontal Cortex of D-Galactose-Treated Rats. Evid-Based Compl. Alt. Med..

[65] Ali W., Ikram M., Park H.Y., Jo M.G., Ullah R., Ahmad S., Abid N.B., Kim M.O. (2020). Oral administration of alpha linoleic acid rescues Aβ-induced glia-mediated neuroinflammation and cognitive dysfunction in C57BL/6N mice. Cells.

[66] Baptista F.I., Henriques A.G., Silva A.M., Wiltfang J., da Cruz e Silva O.A. (2014). Flavonoids as therapeutic compounds targeting key proteins involved in Alzheimer’s disease. ACS Chem. Neurosci..

[67] Schroeter H., Boyd C., Spencer J.P., Williams R.J., Cadenas E., Rice-Evans C. (2002). MAPK signaling in neurodegeneration: Influences of flavonoids and of nitric oxide. Neurobiol. Aging.

[68] Walker E.H., Pacold M.E., Perisic O., Stephens L., Hawkins P.T., Wymann M.P., Williams R.L. (2000). Structural determinants of phosphoinositide 3-kinase inhibition by wortmannin, LY294002, quercetin, myricetin, and staurosporine. Mol. Cell.

[69] Baier A., Szyszka R. (2020). Compounds from Natural Sources as Protein Kinase Inhibitors. Biomolecules.

[70] Ahmad W., Ijaz B., Shabbiri K., Ahmed F., Rehman S. (2017). Oxidative toxicity in diabetes and Alzheimer’s disease: Mechanisms behind ROS/RNS generation. J. Biomed. Sci. Eng..

[71] Adedayo B.C., Jesubowale O.S., Adebayo A.A., Oboh G. (2021). Effect of *Andrographis paniculata* leaves extract on neurobehavioral and biochemical indices in scopolamine-induced amnesic rats. J. Food Biochem..

[72] Kouémou N.E., Taiwe G.S., Moto F.C., Pale S., Ngoupaye G.T., Njapdounke J.S., Nkantchoua G.C., Pahaye D.B., Bum E.N. (2017). Nootropic and neuroprotective effects of Dichrocephala integrifolia on scopolamine mouse model of Alzheimer’s disease. Front. Pharmacol..

[73] Sun K., Bai Y., Zhao R., Guo Z., Su X., Li P., Yang P. (2019). Neuroprotective effects of matrine on scopolamine-induced amnesia via inhibition of AChE/BuChE and oxidative stress. Metab. Brain Dis..

[74] Pattanashetti L.A., Patil B.M., Hegde H.V., Kangle R.P. (2021). Potential ameliorative effect of *Cynodon dactylon* (L.) pers on scopolamine-induced amnesia in rats: Restoration of cholinergic and antioxidant pathways. Ind. J. Pharmacol..

[75] Procházková D., Boušová I., Wilhelmová N. (2011). Antioxidant and prooxidant properties of flavonoids. Fitoterapia.

[76] Moghaddam A.H., Zare M. (2018). Neuroprotective effect of hesperetin and nano-hesperetin on recognition memory impairment and the elevated oxygen stress in rat model of Alzheimer’s disease. Biomed. Pharmacother..

[77] Ishola I.O., Tota S., Adeyemi O.O., Agbaje E.O., Narender T., Shukla R. (2013). Protective effect of *Cnestis ferruginea* and its active constituent on scopolamine-induced memory impairment in mice: A behavioral and biochemical study. Pharm. Biol..

[78] Zhao J., Ren T., Yang M., Zhang Y., Wang Q., Zuo Z. (2020). Reduced systemic exposure and brain uptake of donepezil in rats with scopolamine-induced cognitive impairment. Xenobiotica.

[79] Bruce K.D., Zsombok A., Eckel R.H. (2017). Lipid Processing in the Brain: A Key Regulator of Systemic Metabolism. Front. Endocrinol..

[80] Lesa G.M., Palfreyman M., Hall D.H., Clandinin M.T., Rudolph C., Jorgensen E.M., Schiavo G. (2003). Long chain polyunsaturated fatty acids are required for efficient neurotransmission in *C. elegans*. J. Cell Sci..

[81] Willis L.M., Shukitt-Hale B., Joseph J.A. (2009). Dietary polyunsaturated fatty acids improve cholinergic transmission in the aged brain. Genes Nutr..

[82] Liu Y., Fu X., Lan N., Li S., Zhang J., Wang S., Li C., Shang Y., Huang T., Zhang L. (2014). Luteolin protects against high fat diet-induced cognitive deficits in obesity mice. Behav. Brain Res..

[83] Uriarte-Pueyo I., Calvo M.I. (2011). Flavonoids as acetylcholinesterase inhibitors. Curr. Med. Chem..

[84] Demirci K., Nazıroğlu M., Övey İ.S., Balaban H. (2017). Selenium attenuates apoptosis, inflammation and oxidative stress in the blood and brain of aged rats with scopolamine-induced dementia. Metab. Brain Dis..

[85] Oyama J.-i., Maeda T., Sasaki M., Kozuma K., Ochiai R., Tokimitsu I., Taguchi S., Higuchi Y., Makino N. (2010). Green tea catechins improve human forearm vascular function and have potent anti-inflammatory and anti-apoptotic effects in smokers. Internal. Med..

[86] Xu Y.-Z., Deng X.-H., Bentivoglio M. (2007). Differential response of apoptosis-regulatory Bcl-2 and Bax proteins to an inflammatory challenge in the cerebral cortex and hippocampus of aging mice. Brain Res. Bull..

[87] Kim Y.-J., Kim J.-H., He M.-T., Lee A.-Y., Cho E.-J. (2021). Apigenin Ameliorates Scopolamine-Induced Cognitive Dysfunction and Neuronal Damage in Mice. Molecules.

[88] Li D., Cai C., Liao Y., Wu Q., Ke H., Guo P., Wang Q., Ding B., Fang J., Fang S. (2021). Systems pharmacology approach uncovers the therapeutic mechanism of medicarpin against scopolamine-induced memory loss. Phytomedicine.

[89] Wang R., Shen X., Xing E., Guan L., Xin L. (2013). *Scutellaria baicalensis* stem-leaf total flavonoid reduces neuronal apoptosis induced by amyloid beta-peptide (25–35). Neural Regen. Res..

[90] Cutuli D., Pagani M., Caporali P., Galbusera A., Laricchiuta D., Foti F., Neri C., Spalletta G., Caltagirone C., Petrosini L. (2016). Effects of Omega-3 Fatty Acid Supplementation on Cognitive Functions and Neural Substrates: A Voxel-Based Morphometry Study in Aged Mice. Front. Aging Neurosci..

[91] Ajami M., Eghtesadi S., Razaz J.M., Kalantari N., Habibey R., Nilforoushzadeh M.A., Zarrindast M., Pazoki-Toroudi H. (2011). Expression of Bcl-2 and Bax after hippocampal ischemia in DHA+ EPA treated rats. Neurol. Sci..

[92] Farooq S., Mir S.A., Shah M.A., Manickavasagan A., Mir S.A., Manickavasagan A., Shah M.A. (2022). Chapter 2—Extraction techniques. Plant Extracts: Applications in the Food Industry.

[93] Adusumilli R., Mallick P. (2017). Data conversion with ProteoWizard msConvert. In Proteomics, Humana Press: New York, 2017; 339–368. Proteomics.

[94] Pluskal T., Castillo S., Villar-Briones A., Orešič M. (2010). MZmine 2: Modular framework for processing, visualizing, and analyzing mass spectrometry-based molecular profile data. BMC Bioinform..

[95] Pluskal T., Korf A., Smirnov A., Schmid R., Fallon T.R., Du X., Weng J.-K. (2020). CHAPTER 7 Metabolomics Data Analysis Using MZmine. Processing Metabolomics and Proteomics Data with Open Software: A Practical Guide.

[96] Dührkop K., Fleischauer M., Ludwig M., Aksenov A.A., Melnik A.V., Meusel M., Dorrestein P.C., Rousu J., Böcker S. (2019). SIRIUS 4: Turning tandem mass spectra into metabolite structure information. Nat. Methods.

[97] Aksoz E., Gocmez S.S., Sahin T.D., Aksit D., Aksit H., Utkan T. (2019). The protective effect of metformin in scopolamine-induced learning and memory impairment in rats. Pharmacol. Rep..

[98] Ademosun A.O., Adebayo A.A., Popoola T.V., Oboh G. (2022). Shaddock (Citrus maxima) peels extract restores cognitive function, cholinergic and purinergic enzyme systems in scopolamine-induced amnesic rats. Drug Chem. Toxicol..

[99] Sayed R.H., Ghazy A.H., Yammany M.F.E. (2022). Recombinant human erythropoietin and interferon-beta-1b protect against 3-nitropropionic acid-induced neurotoxicity in rats: Possible role of JAK/STAT signaling pathway. Inflammopharmacology.

[100] Nunez J. (2008). Morris Water Maze Experiment. J. Vis. Exp..

[101] Biggan S.L., Beninger R.J., Cockhill J., Jhamandas K., Boegman R.J. (1991). Quisqualate lesions of rat NBM: Selective effects on working memory in a double Y-maze. Brain Res. Bull..

[102] Mihara M., Uchiyama M. (1978). Determination of malonaldehyde precursor in tissues by thiobarbituric acid test. Anal. Biochem..

[103] Beutler E., Duron O., Kelly B.M. (1963). Improved method for the determination of blood glutathione. J. Lab. Clin. Med..

